# Healthy Eating Index scores associated with symptoms of depression in Cuban-Americans with and without type 2 diabetes: a cross sectional study

**DOI:** 10.1186/1475-2891-10-135

**Published:** 2011-12-09

**Authors:** Joel C Exebio, Gustavo G Zarini, Cristóbal Exebio, Fatma G Huffman

**Affiliations:** 1 Florida International University, Robert Stempel School of Public Health and Social Work, Department of Dietetics and Nutrition, AHC I 449A, 11200 S. W. 8th Street, Miami, FL 33199. USA; 2 Universidad Privada Antenor Orrego, Av. América Sur 3145, Monserrate, Apartado Postal 1075, Trujillo, Perú

## Abstract

**Background:**

Low diet quality and depression symptoms are independently associated with poor glycemic control in subjects with type 2 diabetes (T2D); however, the relationship between them is unclear. The aim of this study was to determine the association between diet quality and symptoms of depression among Cuban-Americans with and without T2D living in South Florida.

**Methods:**

Subjects (n = 356) were recruited from randomly selected mailing list. Diet quality was determined using the Healthy Eating Index-2005 (HEI-05) score. Symptoms of depression were assessed using the Beck Depression Inventory (BDI). Both linear and logistic regression analyses were run to determine whether or not these two variables were related. Symptoms of depression was the dependent variable and independent variables included HEI-05, gender, age, marital status, BMI, education level, A1C, employment status, depression medication, duration of diabetes, and diabetes status. Analysis of covariance was used to test for interactions among variables.

**Results:**

An interaction between diabetes status, gender and HEI-05 was found (*P *= 0.011). Among males with a HEI-05 score ≤ 55.6, those with T2D had a higher mean BDI score than those without T2D (11.6 vs. 6.6 respectively, *P *= 0.028). Among males and females with a HEI-05 score ≤ 55.6, females without T2D had a higher mean BDI score compared to males without T2D (11.0 vs. 6.6 respectively, *P *= 0.012)

**Conclusions:**

Differences in symptoms of depression according to diabetes status and gender are found in Cuban-Americans with low diet quality.

## Background

Type 2 diabetes (T2D) is a major health problem among Hispanics in the United States. The incidence of T2D in Hispanics is twice that of non-Hispanic whites [1]. Previous studies have revealed that the prevalence of depression is higher among individuals with T2D compared to those without diabetes [2,3]. In addition, a relationship between an increase in depression symptoms, retinophaty and nephropathy, have been noted in subjects with diabetes [4]. A study conducted by Black et al. [5] found the effect of the interaction between diabetes and depression to be greater than the sum of the individual effects among older Mexican-Americans, predicting a higher incidence of complications and mortality.

Achieving optimal glycemic control is the goal of the diabetes treatment. Diet is one of the key factors in glycemic control [6], consequently, it affects the physical quality of life of the patient, and it is likely to affect the emotional side as well, specifically feelings and attitudes. In addition, studies have shown that depression is positively associated with poor glycemic control in subjects with T2D [7,8]. Since both, depression and diet quality are independently associated with glycemic control, it is important to determine if these two variables are related for subjects with T2D.

The National Institute of Mental Health suggests that diet may play a major role in depression [9]; however, the research on the effects of nutrition choices on depression symptoms is scarce. For instance, the directionality of this relationship is unclear. It is possible that poor nutritional habits could cause depression or depression may influence poor dietary choices, which have a negative effect on glycemic control in patients with T2D.

Furthermore, the majority of existing research has focused on the effects of individual nutrients on depressive symptoms [10-14]; however, in practice, these findings are difficult to extrapolate due to the interaction of many nutrients in whole foods. Examining overall dietary quality rather than single nutrients may be more practical. The Healthy Eating Index-2005 (HEI-05) is a measure of overall diet quality following federal dietary guidelines, which were established to promote health and reduce the risk of chronic disease [15].

Cuban-Americans represent the third-largest minority group in the United States, of whom two-thirds live in the State of Florida [16]. Approximately 16% of Cuban-Americans ages 45-74 years residing in the United States have diabetes. Although the prevalence of diabetes among Cuban-Americans is lower than Puerto Ricans (26.1%) and Mexican-Americans (23.9%), it is still 1.3 times higher than in non-Hispanic whites. The prevalence of depression measured in the Hispanic Health and Nutrition Examination Survey (HHANES) was 10.2% among Cuban-Americans aged 20-74 years [17]. Cuban-Americans are the least studied of the three largest Hispanic subgroups and few research studies have addressed the areas of depression factors in this population with T2D. This cross sectional study was, therefore, conducted with the aim to determine the association between diet quality, as measured by the HEI-05 score, and symptoms of depression as measured by the Beck Depression Inventory (BDI) among Cuban-Americans living in South Florida.

## Methods

### Participants

Male and female Cuban-Americans with and without T2D were included in a cross sectional study of risk factors for T2D and cardiovascular disease (CVD). Recruitment of participants was conducted in alternate phases of potential subjects with and without T2D from July 2005 to December 2006, age matching subjects by age group. Individuals were initially recruited by random selection (every tenth address) from a randomly generated mailing list of Cuban-American subjects with and without T2D in Miami-Dade and Broward counties, Florida. Letters (10, 000) in English and Spanish, including an invitation flyer, were sent using a pre-purchased list (5000 with, 5000 without diabetes). The list was purchased from Knowledge Base Marketing Inc., Richardson, TX, 75081. Three hundred letters (3%) were returned due to wrong address and 388 (4%) responded to the invitation flyer. The inclusion criteria for subjects with diabetes were self-reported Cuban or Cuban-American ethnicity; age ≥ 30 years; self-report of a medical diagnosis of T2D; able to understand and complete all of the study protocols in English or Spanish; and willing and able to read and sign an informed consent form. Exclusion criteria were pregnant or lactating women, presence of any thyroid disorders and any major psychiatric disorders (not including depression). Subjects without T2D were selected using the same exclusion/inclusion criteria with the exception of the previous diagnosis of diabetes. Initial telephone interview determined previous diagnosis of diabetes, age and gender. Objective of the study was explained, and initial treatment modalities for diabetes were asked. Only 18 subjects did not qualify for the study; for not being Cuban-Americans (n = 2), age < 30 years old (n = 9), and having any other chronic illnesses (n = 7). Eligible participants were invited to participate in a morning session for blood draw and other study related data collection at the Human Nutrition Laboratory, Department of Dietetics and Nutrition, Florida International University. They were instructed to fast for at least 8 hours, avoid smoking and avoid any unusual physical activity prior to the morning session. Fourteen subjects were excluded due to missing values for glycated hemoglobin (A1C, n = 4), duration of diabetes (n = 3), education (n = 3), and energy intake (n = 4). Total data was available for 183 Cuban-Americans with T2D and 173 without T2D which were included in the analysis. A minimum sample size of 122 subjects was previously calculated taking into consideration a maximum of eleven predictors in the model and an effect size of 0.15 for 80% power at a 5% significance level. The study was approved by the Florida International University, Institutional Review board for the inclusion of human subjects.

### Socio-demographic questionnaire

Data were collected using socio-demographic questionnaire, which included questions such as gender, age, education, marital status, employment status, and medication(s) for depression and diabetes. Socio-demographic factors including female gender, age, unmarried, and low educational level have been related to depression in individuals with diabetes in previous studies [2,3,8].

### Anthropometric measurement

Height and weight were measured using a SECA balance scale (Seca Corp, Columbia, MD). Body mass index (BMI) was calculated as weight in kg/height in m^2^. BMI ≥ 30 kg/m^2 ^has been shown to be an independent risk factor associated with depression in individuals with diabetes [8].

### Assessment of depression

Depressive symptoms were measured using the BDI. The BDI is a 21-item, self-reported questionnaire that measures the presence and severity of depressive symptoms using a self-rating scale from 0 to 3 (0 being least depressed and 3 being most depressed). The scale has been previously validated in Hispanics in the United States [18,19]. Scores for each question were summed, calculating a final BDI score. Depression was defined as BDI score ≥ 16. A cut-off point ≥ 16 has shown a specificity of 0.93 and sensitivity of 0.73 [20].

### Assessment of dietary intake and diet-quality scores

Dietary intake was determined using the food frequency questionnaire (FFQ) developed by Willett et al. [21]. This FFQ has been validated in different ethnic groups [21,22] and, also, specifically in Cuban-Americans [23]. Subjects self-reported the average consumption of specific amount of foods over the past 12 months. Frequencies ranged from "never" to "six or more servings per day". The FFQ also assessed the frequency use of multivitamin/mineral supplements, salt, sugar and alcohol. Frequency factors (reported consumption frequencies) of related foods items were summed to calculate the daily servings for each specific food group. The nutrient value of the food item (Harvard University Food Composition Data base, Channing Laboratory, Boston, MA) was multiplied by the frequency of consumption in order to obtain macro and micro nutrient intake.

The HEI-05 score was calculated by converting the daily servings for each specific food into cups or ounces equivalents according to the My Pyramid Equivalent Database version 2.0 [24] and assigning a score according to the following standards. Six components were scored from 0 to 5. Total fruit was assigned 5 points if the intake was equal or more than 0.8 cup equivalents per 1000 kcal. Whole fruit was assigned 5 points if the intake was equal or more than 0.4 cup equivalents per 1000 kcal. Total vegetables received 5 points if the intake was equal or more than 1.1 cup equivalents per 1000 kcal. Dark green, orange vegetables and legumes were allocated 5 points if the intake was equal or greater than 0.4 cup equivalents per 1000 kcal. Total grains received 5 points if intake was equal or greater than 3.0 oz equivalents per 1000 kcal. Whole grains were assigned 5 points if consumption was equal or greater than 1.5 oz equivalents per 1000 kcal. Five components were worth 0-10 points. Milk was given 10 points if intake was equal or greater than 1.3 cup equivalents per 1000 kcal. Meat and beans were allocated 10 points if consumption was equal or higher than 2.5 oz equivalents per 1000 kcal. Oils were assigned 10 points if intake was equal or higher than 12 grams per 1000 kcal. A score of zero was assigned if no items from any particular category were consumed. Proportionally scores were linearly allotted except for saturated fat and sodium. Saturated fat received a score of 10 if intake was less than or equal to 7% of energy, a score of 8 if intake was less than 10% of calories, and zero if intake was equal or greater than 15% of energy. Sodium received 10 points if intake was less than or equal to 0.7 grams per 1000 kcal, 8 points if intake was 1.1 grams per 1000 kcal, and zero if intake was equal or greater than 2.0 grams per 1000 kcal. Calories from solid fat, alcoholic beverages, and added sugars received 20 points if consumption was less or equal to 20% of energy and zero points if intake was equal or greater than 50% of calories. Scores for all 12 components were summed yielding a final HEI-05 score. The overall scale ranged from 0 to 100 points.

### Blood collection

Venous blood (15 ml) was collected from each subject after an overnight fast (at least 8 hours) by a certified phlebotomist using standard laboratory techniques. Specifically, the blood collected was used to test for glycosylated hemoglobin (A1C) (Laboratory Corporation of America (LabCorp^®^). After coagulation, blood was centrifuged at 2500 RPM for 30 minutes. A1C was measured by the DCA2000+ system (Bayer Corporation, Diagnostics Division, N.Y.) using the monoclonal antibody method.

### Statistical analysis

In order to analyze the relationship between diet quality and depression symptoms linear regression with no BDI cut off point and logistic regression using a BDI cut off point of ≥ 16 were run. Depression symptoms was the dependent variable and independent variables included HEI-05 score, gender, age, marital status, BMI, education level, A1C, employment status, depression medication, diabetes status, and duration of diabetes. Since the components of the HEI-05 are energy adjusted on a density basis (per 1000 kcal), the overall score accounted for differences in energy intake; therefore, energy intake (kcal) was not included as a covariate. Initially, a model using only HEI-05 score as the independent variable was run for both the linear and logistic regressions. Several models including the rest of the independent variables were performed. Two and three way interaction terms between HEI-05 score, diabetes status, and gender were included in the models. For the logistic regression, since the USDA did not define specific cut off points for the HEI-05, it was input as a linear variable [15].

Analysis of covariance (ANCOVA) followed by Fisher's least significant difference (LSD) post-hoc test was used to compare mean BDI scores across gender, diabetes status, and median HEI-05 score categories controlling for the previously mentioned covariates. Significance was set at p < 0.05 and all analysis were two sided. Statistical analysis was conducted using SPSS 17.0 (Chicago, IL.).

## Results

Sample characteristics by diabetes status are presented in Table 1. Participants with T2D were older (65.4 ± 11.8 vs. 62.9 ± 11.3, *P *= 0.042) with a higher percentage being males (38.3% vs. 32.9%, *P *= 0.029), had higher BMI (31.6 ± 6.5 vs. 30.0 ± 5.0, *P *= 0.012), unemployed (72.1% vs. 58.4%, *P *= 0.006), not married (50.8% vs. 39.3%, *P *= 0.029), higher A1C (7.6 ± 1.6 vs. 5.9 ± 0.4, *P *< 0.001), BDI score (10.6 ± 10.1 vs. 8.1 ± 6.8, *P *= 0.008), HEI-05 score (57.8 ± 8.9 vs. 53.3 ± 7.8, *P *< 0.001), and lower energy intake (2166.8 ± 795.9 vs. 2358.9 ± 770.2, *P *= 0.021).

**Table 1:** Characteristics of Cuban-Americans by diabetes status in a study of depression symptoms and diet quality

	Cuban Americans		
	
	with type 2 diabetes	without type 2 diabetes	*P *Value
	n = 183	n = 173	
Age (years)	65.4 ± 11.8	62.9 ± 11.3	0.042
Gender (%)			0.029
Male	38.3	32.9	
Female	61.7	67.1	
BMI (Kg/m^2^)	31.6 ± 6.5	30.0 ± 5.0	0.012
Duration of diabetes (years)	9.3 ± 9.5	0	< 0.001
Total calories intake	2166.8 ± 795.9	2358.9 ± 770.2	0.021
Employment status (%)			0.006
Not Working	72.1	58.4	
Working	27.9	41.6	
Marital Status (%)			0.029
Not Married	50.8	39.3	
Married	49.2	60.7	
Education (%)			0.166
< High School	47.0	54.3	
≥ High School	53.0	45.7	
A1C %	7.6 ± 1.6	5.9 ± 0.4	< 0.001
Depression medication (%)			0.719
No	88.5	87.3	
Yes	11.5	12.7	
BDI Scores	10.6 ± 10.1	8.1 ± 6.8	0.008
HEI-05	57.8 ± 8.9	53.3 ± 7.8	< 0.001
HEI-05 median (%)			< 0.001
≤55.6	38.3	61.8	
> 55.6	61.7	38.2	

Data are % or mean ± standard deviation (SD).

BMI = Body mass index, A1C = glycated hemoglobin, HEI-05 = Healthy Eating Index-2005, BDI = Beck Depression Inventory.

Sample characteristics by gender are presented in Table 2. Females had higher BMI (31.4 ± 6.3 vs. 29.9 ± 5.1, *P *= 0.016), not married (54.1 vs. 29.1, *P *< 0.001), depression medication intake (16.6% vs. 3.9%, *P *< 0.001), and HEI-05 score (56.4 ± 8.9 vs. 54.1 ± 8.2, *P *= 0.018) compared to males.

**Table 2:** Characteristics of Cuban-Americans by gender in a study of depression symptoms and diet quality

	Cuban Americans		
	
	Male	Female	*P *Value
	n = 127	n = 229	
Age (years)	63.6 ± 11.0	64.5 ± 12.0	0.524
Diabetes status yes (%)	55.1	49.3	0.296
With type 2 diabetes	55.1	49.3	
Without type 2 diabetes	44.9	50.7	
BMI (Kg/m^2^)	29.9 ± 5.1	31.4 ± 6.3	0.016
Duration of diabetes (years)	5.9 ± 9.7	4.2 ± 7.3	0.090
Total calories intake	2362.3 ± 803.4	2203.6 ± 775.8	0.069
Employment status (%)			0.794
Not Working	64.6	65.9	
Working	35.4	34.1	
Marital Status (%)			< 0.001
Not Married	29.1	54.1	
Married	70.9	45.9	
Education (%)			0.052
< High School	57.5	46.7	
≥ High School	42.5	53.3	
A1C %	6.9 ± 1.5	6.7 ± 1.4	0.261
Depression medications (%)			< 0.001
No	96.1	83.4	
Yes	3.9	16.6	
BDI Scores	8.2 ± 9.1	10.0 ± 8.5	0.068
HEI-05	54.1 ± 8.2	56.4 ± 8.9	0.018
HEI-05 median (%)			0.082
≤ 55.6	55.9	46.3	
> 55.6	44.1	53.7	

Data are % or mean ± standard deviation (SD).

BMI = Body mass index, A1C = glycated hemoglobin, HEI-05 = Healthy Eating Index-2005, BDI = Beck Depression Inventory.

The results of the linear and logistic regressions using only HEI-05 score as the predictor for BDI scores were not significant (*P *= 0.482 and *P *= 0.819 respectively). When the remaining variables (education, age, gender, BMI, A1C, marital status, antidepressant medication, employment status, diabetes status, and known duration of diabetes) were included in the multilinear regression model, only diabetes status and gender were significant predictors of depression (*R*^2 ^= 0.090, *P *= 0.001). For instance, controlling for all other covariates presence of T2D increases the BDI score by 2.8 units. Similarly, controlling for all other covariates, being male reduces the BDI score by 2.3 units.

In the analysis of covariance, an interaction between diabetes status, gender, and median HEI categories was found (*P *= 0.011). Among males with a HEI-05 score ≤55.6, those with T2D had a higher mean BDI score than those without T2D (11.6 vs. 6.6 respectively, *P *= 0.028). Among males and females with a HEI-05 score ≤55.6, females without T2D had a higher mean BDI score compared to males without T2D (11.0 vs. 6.6 respectively, *P *= 0.012).

## Discussion

Both depression and poor diet quality have been independently associated with poor glycemic control in subjects with T2D, increasing diabetes related complications and decreasing quality of life [6-8]. In this particular sample, diet quality was not associated with symptoms of depression based on the regression analysis. Significant differences in symptoms of depression according to diabetes status and gender were found only among those with the lower diet quality.

Our results contradict findings of previous studies that overall dietary quality, rather than individual nutrients, be related to depression. Higher depression symptoms as measured by the Center for Epidemiologic Studies Depression scale (CES-D) score were associated with lower diet quality as defined by the Alternate Healthy Eating Index among Latinos at risk for T2D [25]. Analysis in this study was not adjusted by energy intake.

Jacka et al. [26] examined the relationship between depression and habitual diet in 1046 Australian women using a diet quality score derived from a food frequency questionnaire concluding that a "traditional" dietary pattern characterized by whole grains, fish, meat, fruits and vegetables was associated with lower odds for major depression. However, this association was attenuated when analysis was adjusted by overall energy intake. The authors explained this phenomena stating that overall amount of "bad" food consumed in the diet may be more relevant to depression than the proportion it represents in the overall diet.

A study using the original HEI found that depression was associated with poorer diet quality in women with breast cancer [27]. According to the researchers, data analysis was not adjusted by energy intake because several individual components of the original HEI were expressed as a function of energy. Therefore, the overall score controlled for differences in kilocalories ingested. However, the original HEI scores were positively and significantly correlated with energy intake.

In fact, one of the major differences between the original HEI and the HEI-05 is the inclusion of a density approach (amounts per 1000 kcal of intake) because the original HEI had a tendency to measure quantity rather than quality [15].

In our study, the use of the HEI-05 may be the reason why an association between depression symptoms and diet quality was not found. Subjects with T2D may have better HEI-05 scores because they were eating fewer kilocalories than subjects without T2D. This panorama may have changed if a score not based on 1000 kcal could have been used.

In addition, participants with T2D had a significantly higher BDI score compared to those without T2D, but also, a higher HEI-05 score, meaning that, even thought they had better nutritional habits, they still had more depressive symptoms. This may imply that other factors like gender and diabetes status may be better predictors of depression in this particular sample of Cuban-Americans.

Still our results showed that significant differences among gender and diabetes status are found in participants with the lowest overall diet quality, meaning that special emphasis must be placed in the diets of females and subjects with T2D with symptoms of depression. In addition, since differences are only significant below the median value (HEI-05 ≤ 55.6); this may be an adequate cut off point to divide poor and good diet quality in this population.

The BDI is a practical instrument that can be used in a clinical setting to detect T2D patients with symptoms of depression in order to warrant appropriate nutrition interventions. Nutritional habits are considered environmental factors, which could be changed with adequate nutrition counseling, education and providing related nutritional services, and may have a profound impact in both depression and T2D management.

Limitations of our study included the cross sectional design, which cannot establish causality. In addition, diagnosis of depression was based on a self-reported BDI score and there was no psychiatric or psychological diagnosis of depression. Finally, our low response rate (4%) may indicate that our sample was not representative of the general Cuban-American population and results cannot be generalized. However, this low response rate was expected. Due to their political history, Cuban-Americans are afraid of government control, may be less likely to share information with any organization [28].

## Conclusions

Cuban-American males with T2D and females without T2D with poor overall diet quality had higher symptoms of depression.

## Abbreviations

T2D: type 2 diabetes; HEI-05: Healthy Eating Index-2005; HHANES: Hispanic Health and Nutrition Examination Survey; BMI: body mass index; BDI: Beck Depression Inventory; FFQ: food frequency questionnaire; A1C: glycosylated hemoglobin; CES-D: Center for Epidemiologic Studies Depression Scale.

## Competing interests

The authors declare that they have no competing interests.

## Authors' contributions

JCE analyzed data and prepared original draft, GGZ recruited subjects, collected data and calculated indexes, CE designed research model, conducted statistical analysis and wrote statistical methodology. FGH designed the experiments, received funding, collected data and analyzed data. She also directed the study and manuscript writing. All authors provided comments about the implications of findings and approved the final manuscript version.

## Authors' information

JCE: received a bachelor's degree in Agro-Industrial Engineering from Universidad Nacional de Trujillo, Peru; and a master's degree in Dietetics and Nutrition from Florida International University. He is currently a Teaching/Research Assistant and PhD Candidate in Dietetics and Nutrition, College of Public Health and Social Work at Florida International University in Miami.

GGZ: is a Registered Dietitian (RD) and received his master's degree in Dietetics and Nutrition from the College of Public Health and Social Work at Florida International University (FIU). Currently he is a Research Project Coordinator at FIU where he manages a data collection team in the area of diabetes prevention and ethnicity.

CE: is a Principal Professor in the Department of Health Sciences, School of Medicine at Universidad Privada Antenor Orrego in Trujillo, Peru. Professor Exebio received his bachelor's degree in Statistics from Universidad Nacional de Trujillo, his MS in Quantitative Methods from Universidad Nacional Mayor de San Marcos and Ph.D. in Pedagogy from Universidad Privada Antenor Orrego. He has published several books related to statistics for research and biostatistics.

FGH: is Professor and Chair at Florida International University. She received her PhD from Auburn University in Nutrition and Biochemistry. She is a Registered Dietitian. She is funded by NIH and conducts research in ethnicity and chronic diseases including type 2 diabetes. Her interest in chronic diseases and ethnicity (under studied populations) are published internationally.
